# Tumour-associated lymphangiogenesis in conjunctival malignant melanoma

**DOI:** 10.1136/bjo.2008.147355

**Published:** 2009-07-23

**Authors:** P Zimmermann, T Dietrich, F Bock, F K Horn, C Hofmann-Rummelt, F E Kruse, C Cursiefen

**Affiliations:** 1 Department of Ophthalmology, University Erlangen-Nürnberg, Erlangen, Germany; 2 Department of Ophthalmology, University Medical Center Regensburg, Regensburg, Germany

## Abstract

**Background:**

To evaluate whether tumour-associated lymphangiogenesis, that is the formation of new lymphatic vessels (LVs) induced by a tumour, occurs in and around conjunctival malignant melanoma (MM).

**Methods:**

Clinical files and conjunctival specimens of 20 patients with histologically diagnosed conjunctival MM were analysed. Sections were stained with LYVE-1 and podoplanin antibodies as specific lymphatic endothelial markers and Ki67 as proliferation marker. The tumour area and the area covered by LV (LVA), LV number (LVN) and LV density (LVD) were measured within the tumour and in the peritumoural area in digital images of the specimen. The LV results were correlated with the histopathological characteristics, tumour location, recurrence rate, mitomycin C therapy and presence of metastases.

**Results:**

LVs were detected in all specimens within the tumour and peritumourally. Significantly more Ki67^+^ proliferating lymphatic endothelial cells were detected in the tumour and in the peritumoural tissue up to 300 μm compared with the surrounding normal conjunctiva (>300 μm distance). There was a slightly positive correlation between the tumour size and the LVN and LVA in the 50 μm zone adjacent to the tumour. We did not find any significant correlations between LVs and histopathological and clinical characteristics (location, shape, relapses, metastases), possibly due to the small sample sizes. Non-limbal tumours with involvement of tarsus or fornix showed a tendency towards a higher LVD compared with limbal tumours.

**Conclusion:**

Conjunctival MMs display tumour-associated LV within and around the tumour. The MM seems to induce lymphangiogenesis not only in the tumour, but also in its proximity.

Malignant melanomas (MMs) of the conjunctiva are associated with significant morbidity and mortality due to high rates of recurrence and metastasis.^1^ ^2^ The dissemination of the tumour is linked to regional lymph nodes with subsequent distant metastasis.^3^ Compared with cutaneous MM, conjunctival MM is rare. The annual age-adjusted incidence rates (per million) vary from 0.15 in Asians to 0.5 in non-Hispanic Caucasians.^4^ ^5^

To date, only a few features have been recognised as prognostic factors for conjunctival MM: tumour location, expansion, relapse, multifocal location, involvement of the surgical margins and tumour depth are known prognostic factors for metastatic disease.^6^ ^7^ Histopathological characteristics seem not to be consistently associated with the clinical outcome.^7^

The primary treatment of conjunctival MM is surgical: complete excision with tumour-cell free margins represents the therapy of choice but cannot be sufficiently performed in cases of diffuse growth. Topical mitomycin C as adjunct therapy has been established,^8^ and cryotherapy, laser ablation, radiation treatment and chemotherapy in case of metastasis represent additional treatment options for conjunctival MM.

Conjunctival MMs are rich in blood vessels, which play a role in systemic haematogenous metastasis. However, the main route of metastasis of conjunctival MM is lymphogenic: ultrasonic examination of the draining lymph nodes or even surgical removal of the sentinel lymph nodes has been recommended. Up to now, it was not known whether conjunctival MMs also display significant tumour-associated lymphangiogenesis, that is whether the tumour induces the formation of new lymphatic vessels.

The extent of lymph node metastasis is supposed to be a major determinant for prognosis and staging of tumours,^9^ and it has been shown that tumour-induced lymphangiogenesis is a strong risk factor for tumour metastasis in different human cancers.^3^ ^9^ ^10^ ^11^ ^12^ ^13^ ^14^ The importance of tumour-induced lymphangiogenesis for lymphogenic metastasis in cutaneous MM has been shown recently.^10^

The purpose of this study was to determine whether conjunctival MMs also display tumour-induced lymphangiogenesis, which may represent a possible new prognostic factor. We used specific lymphatic endothelial markers to analyse the presence of lymphatic vessels (LVs) in the tumour itself and in the adjacent tissue, and correlated these data with the clinical outcome and histopathological characteristics of the tumours.

## Material and methods

### Patients and conjunctival sections

Clinical files and histological sections of conjunctival MMs of 20 patients who were treated at the Department of Ophthalmology of the University Erlangen-Nürnberg, Germany, between 1987 and 2005, were analysed retrospectively.

The files were screened, and the documented treatment and follow-up were taken into consideration. The clinical outcome of all patients was re-evaluated at the end of 2006 and again in 2008 by interviewing the patients’ general practitioners for any new progress of the disease since the last visit, especially for systemic metastasis.

### LV staining (LYVE-1 and podoplanin)

For staining of LVs, LYVE-1 served as a specific marker for lymphatic vascular endothelium. The preparation of the histological sections of conjunctival MMs was performed as described previously.^15^ Briefly, tissue was fixed in neutral buffered formalin, embedded in paraffin and cut in 4 μm sections. After deparaffinisation and rehydration, sections were digested with proteinase K (Dako, Hamburg, Germany) and incubated for 10 min with horseradish peroxidase (HRP). Sections of conjunctival MMs were incubated for 30 min with a rabbit polyclonal antibody against human LYVE-1 (1:100; Dako, Hamburg, Germany) and HRP-conjugated secondary antibody before development with 3-amino-9-ethylcarbazole (AEC^+^) substrate (red reaction product) or 3,3′-diaminobenzidine (DAB; brown product). Sections were counterstained with Mayer haemalaun (Chroma, Münster, Germany). Positive controls were performed on corneoscleral ring specimens and negative controls with control IgG.

Since LYVE-1 is also expressed on tissue macrophages,^16^ ^17^ only clearly identifiable vessels with an erythrocyte-free vessel lumen were counted as LV, and specimens were double-stained with podoplanin as a second lymphatic endothelial marker.

For podoplanin immunostaining, polyclonal rabbit antihuman antibody against podoplanin (1:200, Dako, Hamburg, Germany) was used, followed by biotinylated goat antirabbit IgG for 30 min and detection by a streptavidin peroxidase complex (using DAB/AEC^+^ as the chromogen substrate). Positive controls were performed as described above.

### Ki67 staining

Sections of paraffin embedded specimens were double-stained with LYVE-1 and monoclonal antibody against Ki67 (clone MIB-1, Dako, Hamburg, Germany) as a specific marker for proliferating cells. LVs with at least five endothelial cells with nuclear Ki67 positivity were considered to be Ki67-positive.

### Mitomycin C treatment

The additional topical mitomycin C treatment of conjunctival MM by eye-drops is standardised in our department as two 14-day cycles with mitomycin C 0.02% eye-drops five times a day with a 14-day break. Some patients were not treated with mitomycin C eye-drops due to allergy or refusal. To analyse the potential antilymphangiogenic effect of mitomycin C therapy, tumour specimens of patients who received mitomycin C treatment and had excisions later on during their clinical course (because of new suspect lesions) were compared with the specimens obtained before mitomycin C treatment.

### Microscopy and computer-assisted vessel analysis

Histological sections of conjunctival MMs of 20 patients were taken into consideration. Sections were analysed with a light microscope (BX51, Olympus Optical Co., Hamburg, Germany), and digital colour images were taken with a 12-bit CCD camera (Color-View I, Olympus, Hamburg, Germany; 40× and 100× magnification). Analyses were performed using CellˆF (Olympus, Hamburg, Germany) and Image J analysing program (available via http://rsb.info.nih.gov/ij/download.html). Morphometric LV analysis was performed for the area of the tumour, the adjacent 50 μm zone, the mid-peripheral zone (50–200 μm), the peripheral zone (200–300 μm) and the conjunctiva more than 300 μm away from the tumour border (defined as normal conjunctiva). If the tumour-adjacent area was not completely represented on the specimen, we evaluated the area as far as represented. The tumour size was measured as the area covered by the tumour in the histological section. We determined the following parameters: (1) the LV number (LVN), (2) the area covered by LVs (LVA), (3) the LV density (LVD), determined by measuring the LVN and dividing it by the tumour cross-sectional area (mm^2^).

### Functional and statistical analysis

To determine statistical significance, quantitative analyses of the LVA, LVN and LVD in all analysed areas (intratumoural, 50 μm, 50–200 μm, 200–300 μm and >300 μm peritumoural) were performed in a standardised procedure using the statistic program InStat 3 (GraphPad Software, San Diego, California). Analyses were performed using the non-parametric test for the Ki67 analysis and the Pearson rank correlation for the correlation of tumour area to LVN and LVA.

## Results

### Patients and histopathological characteristics

The median age of the patients in the study was 70.4 years (43–100 years). Nine women and 11 men were treated. The MM of 13 patients was based on primary acquired melanosis (PAM); in seven patients, the origin of the MM remained unclear. The primary treatment was surgical, and 10 patients had an additional topical mitomycin C treatment. The primary tumour was located in the fornix (two patients), the tarsus and the upper lid (five patients) or at the limbus/epibulbar conjunctiva (seven patients). Six patients showed a widely disseminated tumour, including some who have had primary excision outside our department, so that the primary tumour location was not known. Ten patients showed a diffuse, five a nodular and five a mixed growing type of the MM. The histopathological characteristics were: 13 tumours of mixed cell type, five tumours of spindle cell type and two tumours of epitheloid cell type.

Eight of the 20 patients showed more than five relapses during the clinical course. Five patients suffered from metastasis: one patient was diagnosed for gastric metastasis 7 years after primary diagnosis, two patients for submandibular and neck spreading after 2 and 3 years, and one patient for craniopharyngeal metastasis after 1 year, and one patient had parotical metastasis after 14 years.

### Conjunctival MMs display intra- and peritumoural LVs

**Figure 1:**
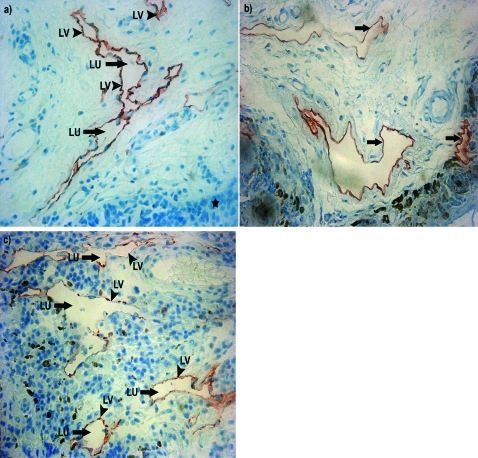
Tumour-associated lymphatic vessels (LVs) in malignant melanomas (MMs) of the conjunctiva. (A) Representative image of LV staining with LYVE-1 antibody as specific marker for lymphatic endothelium: LYVE-1 positive peritumoural LVs. (B) Representative image of podoplanin stained LVs in the tumour adjacent conjunctiva. (C) Intratumoural LYVE-1 positive LVs (magnification ×100/×200). The area of primary acquired melanosis is marked with an asterisk. Arrows denote the LYVE-1/podoplanin stained lymphatic vessels. Note that erythrocyte-filled blood vessels are not stained with these lymphatic endothelial specific markers. LU, lymphatic vessel lumen.

Using LYVE-1 and podoplanin staining, we identified LVs in all included MM specimens, both within the tumour itself and in the adjacent tissue (fig 1). There was a similar staining pattern for both lymphatic vascular endothelial markers in the conjunctival MM specimens.

### Conjunctival MMs are associated with intra- and peritumoural lymphangiogenesis

**Figure 2:**
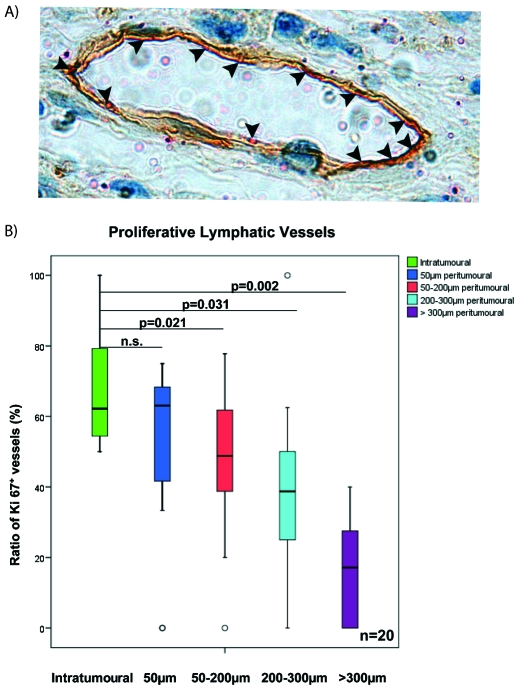
Tumour-induced lymphangiogenesis. Significantly more proliferating lymphatic vessels (LVs) were found intratumourally and next to the tumour than in distant conjunctiva (>300 μm). Representative images of conjunctival malignant melanoma specimen stained with LYVE-1 and proliferation marker Ki67. Ki67 positive cells are marked (arrowhead). (A) Representative image of Ki67 positivity in tumour-associated lymphatic endothelial cells; red: Ki-67; brown: LYVE-1 (magnification ×1000). (B) Significantly more proliferating LVs were found intratumourally as well as in the directly adjacent tumour environment than in the more distant conjunctiva. A paired t test was performed for each zone separately with the following results: (1) tumour versus tumour adjacent conjunctiva (50 μm zone) p = 0.063 (not significant); (2) tumour versus mid-peripheral zone (50–200 μm) p = 0.021; (3) tumour versus peripheral zone (200–300 μm) p = 0.031; (4) tumour versus conjunctiva >300 μm from the tumour border p = 0.002; n = 20, median is marked; circles mark three outliers. The results support the hypothesis of tumour-associated active lymphangiogenesis in the proximity of the tumour.

To examine whether conjunctival MMs induce formation of new LVs, Ki67 staining was performed to detect proliferating lymphatic endothelial cells in the conjunctival MMs and the adjacent conjunctival tissue. Immunostaining with Ki67 revealed significantly more proliferating lymphatic endothelial cells in the tumour and in the directly adjacent conjunctiva compared with the peripheral zones. Non-parametric tests were performed for each zone separately with the following results concerning the ratio of Ki67-positive LVs: (1) tumour versus tumour adjacent conjunctiva (50 μm zone) p = 0.063 (not significant); (2) tumour versus mid-peripheral zone (50–200 μm) p = 0.021; (3) tumour versus peripheral zone (200–300 μm) p = 0.031; (4) tumour versus distant, presumably normal conjunctiva >300 μm from the tumour border p = 0.002 (fig 2). The results support the hypothesis of tumour-associated active lymphangiogenesis in the tumour and its proximity.

### Influence of tumour cross-sectional area on LVs

**Figure 3:**
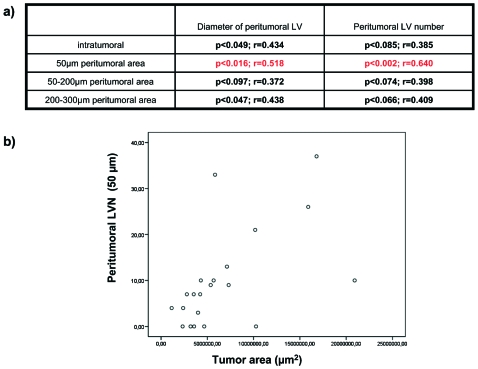
Influence of tumour cross-sectional area on lymphatic vessels (LVs). Analyses of the LV parameters area covered by LV (LVA), LV number (LVN) and LV density (LVD) were performed in the tumour and in the tumour environment (50 μm zone, 50–200 μm zone, 200–300 μm zone). Intratumoural LVN, LVA and LVD were not positively correlated to the tumour cross-sectional area (A). There was a slightly inverse correlation for the LVD in the tumour with the tumour cross-sectional area (r = −0.257, p = 0.237). The LVN in the 50 μm area directly adjacent to the tumour was positively correlated to the tumour cross-sectional area (Pearson correlation coefficient r = 0.64; p = 0.002), demonstrated as a scatter plot (B).

We analysed whether the extent of lymphangiogenesis in and around conjunctival MM was correlated with the tumour area. Therefore, analyses of the LV parameters LVA, LVN and LVD were performed in the tumour and in the tumour environment (50 μm zone, 50–200 μm zone, 200–300 μm zone). Intratumoural LVN, LVA and LVD were not positively correlated to the tumour cross-sectional area (results for LVN and LVA shown as table in fig 3). The LVN and LVA in the 50 μm area directly adjacent to the tumour are positively correlated to the tumour cross-sectional area; the results for the LVN are demonstrated as a scatter plot in fig 3 (Pearson correlation coefficient r = 0.64; p = 0.002). There was a slightly inverse correlation for the LVD in the tumour with the tumour cross-sectional area (r = −0.257, p = 0.237).

### Effect of mitomycin C therapy on LVs

We analysed the potential effect of topical mitomycin C treatment on LV formation.

**Figure 4:**
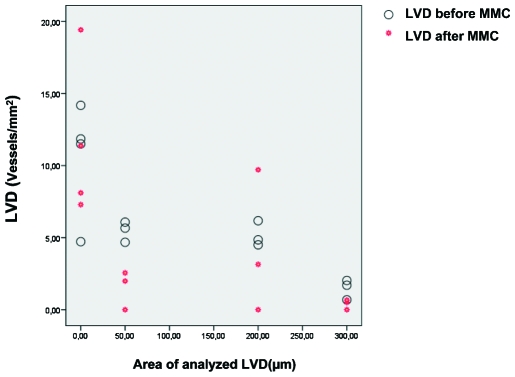
Effect of topical mitomycin C on lymphatic vessels (LVs): scatter plot of the measured LV density (LVD) in the tumour (0), in the adjacent 50 μm zone (50), in the 50–200 μm zone (200) and in the 200–300 μm zone (300) before and after mitomycin C therapy. LVD before mitomycin C therapy is marked as a ring, and LVD after mitomycin C therapy is marked as an asterisk. Sections of four patients were analysed, in one patient there was no surrounding conjunctiva represented on the specimen, and only the tumour area was analysed. Because of the limited number of patients, descriptive analyses were performed.

Specimens of patients (n = 4) who underwent topical mitomycin C treatment after tumour excision and had subsequent excisions for tumour recurrence were analysed for LVD and LVN (fig 4). In three patients, the specimens represented all five zones; in one patient, the specimens showed only a tumour without surrounding conjunctiva because of diffuse tumour growth (therefore only the tumour zone was analysed in this patient). Because of the limited number of patients, the statistical analysis was restricted to descriptive analyses (fig 4). An additional LV analysis (LVD, LVN, LVA) of specimens from mitomycin C treated patients compared with specimens from patients without mitomycin C treatment did not show any statistically significant results.

### Analysis of LVs in relation to tumour location

**Figure 5:**
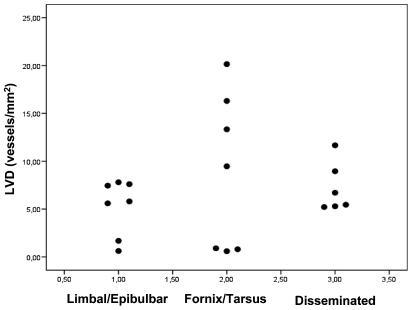
Analysis of lymphatic vessels (LVs) in relation to tumour location. Descriptive scatter plot: in four of seven patients with non-limbal malignant melanomas (MMs) with involvement of the fornix or tarsus, the LV density (LVD) was higher than in all limbal/epibulbar tumour specimens (n = 7). Seven patients had limbal/epibulbar MMs, seven patients had an MM at the fornix or tarsus, and six patients had a disseminated MM. The analyses of LV parameters (LV number (LVN), area covered by LV and LVD) did not show any statistically significant results in these small sample sizes.

Analysing the location of the tumour, seven patients had limbal/epibulbar tumours, seven patients had a MM at the fornix or tarsus, and six patients had a disseminated MM. The analyses of LV parameters (LVN, LVA, LVD) did not show statistically significant results in these small sample sizes. Because of the small number of patients, we performed only descriptive analyses as a scatter plot. Figure 5 demonstrates the LVD in relation to the tumour location. In four of seven patients with non-limbal MMs with involvement of the fornix or tarsus, the LVD was higher than in all limbal tumour specimens (n = 7).

## Discussion

This study on conjunctival MM shows for the first time that conjunctival—and not only cutaneous—MMs display tumour-associated lymphangiogenesis.

In our study, we found LVs in the tumour itself as well as in the peritumoural area. These erythrocyte-free LVs are stained with two new markers specific for lymphatic vascular endothelium, which are LYVE-1 and podoplanin. Most remarkably, the degree of actively proliferating Ki67 positive lymphatic vascular endothelial cells is significantly higher within the MM and in the close vicinity of the tumour compared with normal, resting conjunctival LVs more distant from the tumour site. The ratio of Ki67 positive LVs is reduced with growing distance from the tumour border, which supports the hypothesis of active tumour-induced formation of new LVs (tumour-associated lymphangiogenesis). It has been shown recently that lymphangiogenic growth factors, which are secreted by a primary tumour, can induce lymphangiogenesis.^20^ ^21^ ^22^ ^23^

In our small pilot study, there was no significant correlation between tumour size (two-dimensional) and LVN and LVA in the tumour. The analyses of the tumour environment revealed a higher LVN and LVA in the 50 μm zone directly adjacent to the tumour, possibly being an indicator for active lymphangiogenesis in the tumour surroundings. We found a slightly inverse correlation between LVD in the tumour and the tumour cross-sectional area. On the one hand, this finding might be related to the two-dimensional calculations of the tumour area based on cross-sectional specimens of the tumour. On the other hand, small tumours might show a higher LVD as a signal for their starting potency of dissemination, possibly related to high secretion rates of lymphangiogenic growth factors. Another possible aspect is a centrally developing necrosis in larger tumours which might reduce the rate of lymphangiogenesis and cause reduced LVD. Furthermore, some studies describe a high interstitial pressure within tumours that promotes LV collapse;^13^ thus, compressed LVs in larger tumours might appear smaller than LVs in smaller tumours.

We analysed the putative impact of lymphangiogenesis on recurrence rate and metastasis but did not find any significant correlation, possibly related to the small sample sizes. Larger (prospective) studies now will have to evaluate tumour-associated lymphangiogenesis as a putative risk factor for tumour metastasis.

Tumour location is one of the clinically most important prognostic predictors of conjunctival MM: non-limbal tumours show a higher incidence of initial systemic metastasis and reduced survival rates.^5^ ^7^ This fact might be due to facilitated access to blood vessels or to the draining LVs Our descriptive analysis of the tumour location, that is limbal versus palpebral/fornix versus disseminated conjunctival in correlation with parameters of lymphangiogenesis, showed a tendency of higher LVD in non-limbal palpebral/fornix tumours. We did not find any significant differences concerning the lymphangiogenic parameters in correlation with the different growth patterns, that is the shape of the conjunctival MM. Additional studies are necessary to elucidate these aspects.

Topical mitomycin C treatment might provide an antilymphangiogenic effect, as has been suggested in several other studies on the treatment of conjunctival MM.^8^ ^18^ We performed only descriptive analyses due to the small sample sizes of histological tumour specimens after mitomycin C treatment: there was a tendency of reduced LVD in specimens from patients after mitomycin C therapy compared with specimens before mitomycin C therapy, while LVN and LVA were not reduced. Nevertheless, other factors such as fibrotic tissue-remodelling after surgical excision, the influence of co-medications as topical steroids or other causes cannot be ruled out and may have a significant influence.

The prognostic importance of intra- and peritumoural lymphangiogenesis is becoming more established by a growing number of studies on several human cancers.^3^ ^9^ ^10^ The identification of high-risk patients is helpful in order to individually optimise screening and treatment guidelines. The extent of tumour-associated lymphangiogenesis in conjunctival MM may be one novel prognostic criterion besides other known or putative risk factors. Immunostaining for lymphatic markers such as LYVE-1 and podoplanin in routine histological work-up of tumour samples might be warranted if future studies reveal a correlation between tumour-induced lymphangiogenesis and prognosis of the tumour in terms of metastasis and recurrence rate. The histological analysis of lymphangiogenesis parameters compared with sentinel lymph node biopsy as a mean of guiding treatment and follow-up is under discussion.^9^ ^12^ ^19^

This study on tumour-associated lymphangiogenesis may pave the road to new targets for innovative therapeutic approaches and serve as a novel prognostic parameter in conjunctival MM. Much effort in antilymphangiogenic and antiangiogenic research has been made to develop new therapeutic approaches to inhibit tumour spreading. Recently, different assays of selective inhibition of LV growth on the ocular surface have been shown.^24^ ^25^ In the future, antilymphangiogenic treatment options might help to minimise the risk of metastasis in conjunctival MM.

## Conclusion

MMs of the conjunctiva display LVs within and around the tumour. There is evidence for tumour-induced lymphangiogenesis, that is formation of newly formed LVs. These LVs may act as conduits for tumour metastasis. Lymphangiogenesis parameters such as LVD and LVN may become useful novel prognostic indicators for conjunctival MM. Novel antilymphangiogenic therapeutic strategies may help to optimise the therapy of conjunctival MM in the future.
